# Practical Medicine

**Published:** 1878-10

**Authors:** 


### Practical Medicine.

Fatal Purpura Following the Use of Iodide of Potassium.—Mackenzie.—(*Br. Med. Jour.*, June 8, 1878.)

An infant, aged five months, had prescribed for it a mixture containing 0.15 of potassic iodide at a dose, for congenital syphilis. Three quarters of an hour after taking the first dose the child's face was noticed to "turn black." The extravasations rapidly and visibly increased in size, the eyelids closed, the face and chin were tensely swollen, a few spots appeared on the arms and legs, and after sixty-eight hours the babe was dead.

The autopsy showed necrosis of the purpuric spots on the face, and syphilis and ulceration of the intestines. But a single dose had been taken.

The author thought it would be necessary to review what was known of syphilitic and iodic purpura. His opinion of this case was that while there was undoubted syphilitic dyscrasia; the fatal purpura was due to the iodide.

